# Knowledge and Awareness of Parents About the Difference Between Attention Deficit Hyperactivity Disorder and Childhood Absence Epilepsy in the Paediatric Population Makkah, Saudi Arabia: A Cross-Sectional Study

**DOI:** 10.7759/cureus.37945

**Published:** 2023-04-21

**Authors:** Rahaf S Alotaibi, Ghadi A Alghamdi, Anas Alloqmani, Nada S Almuntashiri, Khaled a alharbi, Jamil A Samkari, Abdullah A Tawakul, Omar Babateen

**Affiliations:** 1 Medicine, Umm Al-Qura University, Makkah, SAU; 2 Family and Community Medicine, King Abdulaziz University Faculty of Medicine, Jeddah, SAU; 3 Neurology, Umm Al-Qura University, Makkah, SAU; 4 Surgery, Umm Al-Qura University, Makkah, SAU

**Keywords:** childhood absence epilepsy (cae), makkah, saudi arabia, level of awareness, attention deficit hyperactivity disorder (adhd)

## Abstract

Introduction: Attention deficit hyperactivity disorder (ADHD) is a neurodevelopmental disorder, mainly in children. The signs and symptoms of ADHD include inattention, impulsivity, and hyperactivity. Consequently, Childhood Absence Epilepsy (CAE) tends to present in children with sudden and recurrent episodes of loss of awareness alongside symptoms that occasionally include clonic, atonic, and simple automatisms. The present study evaluates parents' knowledge in Makkah regarding the difference between ADHD and CAE.

Methodology: The study was conducted among Saudi Arabian parents living in Makkah. Data were collected in April 2022 through the use of an online survey that was distributed electronically via social media platforms. The inclusion criteria entailed parents from different socio-economic backgrounds. In contrast, the exclusion criteria entailed parents who had not been involved in raising their children and those with children with intellectual disabilities. A group of consultants was tasked with validating all data collected through an original questionnaire. To effectively calculate the study sample size, OpenEpi Version 3.01 was used. Lastly, all statistical analyses were conducted with Stata Social Sciences (SPSS®) software for Mac, version 26 (IBM Corp., Armonk, NY, USA).

Results: A total of 633 participants completed the survey. Of the total respondents, approximately 1% indicated having a good knowledge level, 15.17% indicated having moderate knowledge, and the remaining 84% indicated poor knowledge of the subject under study. Approximately 46% of the participants reported that social media was the primary source of information. One significant issue regards the observation that the parent's level of education was statistically associated with the level of knowledge.

Conclusion: There is limited awareness of the difference between (ADHD) and (CAE) among parents in the pediatric population. These findings highlight an opportunity to raise awareness using well-organized education programs in Makkah City.

## Introduction

Attention deficit hyperactivity disorder (ADHD) is a common neurodevelopmental disorder that affects children and often continues into adulthood. The symptoms of ADHD include patients experiencing difficulties paying attention and the inability to control impulsive behaviors by acting without thinking about the consequences or hyperactivity [1]. The present study was conducted in Saudi Arabia, and data was collected from 708 primary school pupils attending grades 1-3 (7-9 years old) by teachers and parents in 2007. The study findings indicated that 2.7% of the children have ADHD [2].

Consequently, CAE refers to the sudden and recurrent episodes of loss of awareness infrequently accompanied by clonic, atonic, or simple automatisms or autonomic components. Two types of CAE exist, namely, typical and atypical. Approximately 10% of children with epilepsy tend to have typical CAE. The prevalence rate of CAE in the general population is 5 to 50/per 100,000. Comparable figures have been disclosed by studies conducted in the US (Connecticut) and Europe-based (Scandinavia, France) [3].

As previously mentioned, CAE and ADHD are two completely different disorders. For instance, the observed differences include the view that in the CAE, the episodes are always accompanied by eyelid fluttering and jerky muscle movement [4]. In ADHD, the patients do not portray motor symptoms [5]. However, there are also similarities between ADHD and CAE, including the observation that the two conditions negatively influence students' school performance, attention, and cognitive abilities [6,7]. Considering that parents are the principal caregiver of their children and their knowledge regarding their medical history and well-being is vital to providing them with the most suitable management, the parents' ability to differentiate between ADHD and CAE is critical. As such, the present study's aims include an evaluation of the current knowledge of parents in Makkah about ADHD and CAE, as well as to understand the cardinal difference between ADHD and CAE and their sources of information.

This article was previously posted to the 18th PAN Arab & 13th GCC & 26th SNS conferences on January 12-14, 2023.

## Materials and methods

Study population and sampling methodology

The present study is a community-based cross-sectional descriptive research conducted in Makkah City, Saudi Arabia, among parents living there. Parents from different socioeconomic statuses, both males and females, who agreed to partake in the study were included. Further, participants who were not involved in raising their children and parents with children suffering from intellectual disabilities were excluded. Data were collected in April 2022 using an original online questionnaire that a selected group of consultants validated. The survey was formulated in Arabic and English and completed using Google Forms. The survey was also distributed electronically through various social media platforms. A total of 632 participants completed and delivered the study.

The questionnaire was organized into sections that included the participants' sociodemographic data, the assessment of the participant's knowledge of ADHD and CAE, and the participants' source of information about both conditions. The responses from the participants were collected as either yes or no, multiple choice, and written answers. Still, the knowledge levels were categorized as a poor level of knowledge (0-60%), moderate knowledge level (60 - 79%), and good knowledge level (80 - 100%). The original questionnaire used in this study is provided in the appendix section.

Data analysis

For the present study, data were analyzed using the Statistical Package for Social Sciences (SPSS®) software for Mac, version 26 (IBM Corp., Armonk, NY, USA). To analyze the numerical variables, the authors used the t-test and chi-square test in analyzing the categorical data. The 95% confidence intervals and P-values were reported for every variable included. Moreover, the present study's P-value was < 0.05 for statistical significance. 

Ethical considerations

For the present study, ethical approval was provided by the Institutional Review Board (IRB) of UQU (No. HAPO-02-K-012-2022-04-1049). Further, consent was obtained electronically from all participants following an explanation of the study's aims and objectives.

## Results

Demographics

A total of 632 study participants completed and returned their questionnaires. Females formed the most significant proportion of the participants at 68.2%. Most of the study participants, 33.86%, were aged between 18 and 28. Concerning the participants' place of residence, 82.75% reported that they were residing in Makkah. Still, 85.13% of the participants reported being married, even as 67.25% noted they held a bachelor's degree. A more significant proportion of the participants, 42.25%, were employed even as 31.06% reported having incomes ranging between 5000 and 10000SR. Table 1 below shows the demographic attributes of the study population.

**Table 1:** Demographic data

Demographic characteristics of the responders		
	Freq	Percent%
Age category		
18-28	214	33.86
29 - 39	160	25.32
40 - 50	169	26.74
51-61	79	12.5
62 and above	10	1.58
Gender		
Female	431	68.2
Male	201	31.8
Residence		
Mecca	523	82.75
Outside Mecca	109	17.25
Education		
Bachelor's degree	425	67.25
General high school / middle school	142	22.47
Master's degree	58	9.18
illiterate	7	1.11
Marital status		
Divorced	43	6.8
Married	538	85.13
Widow	51	8.07
Income		
10000 - 15000 SR	163	25.83
5000 - 10000 SR	196	31.06
Less than 5000 SR	117	18.54
More than 15000 SR	155	24.56
Employment status		
Unemployed	63	9.97
Employed	267	42.25
Retired	43	6.8
Student	127	20.09
Unemployed	132	20.89

Awareness levels

The awareness levels of CAE and ADHD were divided into three categories: good, moderate, and poor.

Gender 

The study's findings indicated that, of the female participants, 82.59% had poor awareness levels, 16.47% showed moderate awareness levels, and 0.92% portrayed good awareness levels. None of the male participants' groups showed an excellent awareness level, even as 12.43% showed a moderate awareness level and 87.56% showed poor awareness.

Social status

Regarding marital status, of the divorced participants, 0.55% indicated good knowledge and awareness levels, 16.35% indicated moderate knowledge and awareness, and 83.08% indicated poor knowledge and awareness levels. Consequently, among the married participants, none indicated good familiarity and awareness levels, 9.3% indicated having moderate knowledge and awareness levels, and 90.69% indicated poor knowledge and awareness levels. Further, 1.96% of the widows' participants had good knowledge and awareness levels, 7.84% had moderate knowledge, and 90.19% had poor knowledge and awareness. The P value was 0.237. Nevertheless, the overall results showed that 0.79% presented good familiarity and awareness levels even as 15.17% had moderate knowledge and awareness levels, and 84.4% had poor knowledge and awareness levels. 

Age category 

The number of participants who displayed a poor level of awareness was highest among participants aged between 18 and 28 years at 85.51%. Consequently, 13.55% and 0.93% of the participants showed moderate and reasonable levels of awareness, respectively. Similar results regarding participants aged 29 to 39 were observed, given that 0.62% indicated good awareness levels, 15.62% showed moderate awareness, and 83.75% displayed poor awareness. A more significant proportion of participants aged between 40 and 50 indicated an insufficient level of awareness at 84.02%. In comparison, a small proportion portrayed a moderate level of understanding at 15.38%, and 0.59% portrayed an excellent level of awareness. None of the participants aged between 51 and 61 indicated a good level of understanding, even as 17.72% of them portrayed moderate awareness levels and 82.27% indicated poor awareness levels. Additionally, none of the participants above 62 years of age had a good level of awareness, even as 20% showed moderate awareness levels, and the remaining 80% indicated poor awareness levels.

Home residence

Regarding residency awareness among individuals residing in Makkah, the results indicated no participant had good awareness levels. In contrast, 11.92% of the participants portrayed moderate knowledge levels, and 88.07% had poor knowledge levels. However, among the participants residing outside Makkah, 0.76% had good knowledge levels, 15.86% had moderate knowledge levels, and 83.36% had poor knowledge levels. The p-value was 0.369.

Educational level

The highest proportion of participants who displayed a poor awareness level included those with general high school/middle school education at 85.17%, followed by those bachelor's degree education level at 85.91%, those with master's degree and above education level at 72.41%, and lastly the illiterate at 85.71%. Also, the highest proportion of participants who displayed a moderate awareness level was general high school/middle school level of education at 14.58%, followed by bachelor's degree level of education at 13.38%, master's degree and above level of education at 24.13%, and the illiterate at 14.28%. However, participants with master's degrees education level presented the highest rate of a good level of awareness at 3.44%, which was followed by bachelor's degree holders at 0.73%, general high school/middle school level of education at 0.23%, and the illiterate at 0%. The above result was analyzed and found to be statistically significant (P < 0.045) 

Income and employment status

Regarding the existing correlation between income and awareness levels, it was noted that 0.85% of participants who were employed with incomes that ranged from 10000 to15000 SR presented good knowledge and awareness levels, 9.4% of them presented moderate knowledge and awareness levels, and 89.74% presented poor knowledge and awareness levels. Consequently, regarding the employed participants with incomes ranging from 5000 to 10000 SR, it was noted that 0.51% presented good knowledge and awareness level, 15.81% showed moderate knowledge and awareness level, and 83.67% showed poor knowledge and awareness level. Nevertheless, regarding participants whose earnings were below 5000 SR, it was noted that 1.22% presented good familiarity and awareness levels, 17.17% showed moderate knowledge and awareness level, and 81.59% showed poor knowledge and awareness level. Although none of the participants earning incomes above 15,000 SR presented good familiarity and awareness levels, 16.77% portrayed moderate knowledge and awareness level, while 83.22% showed poor knowledge and awareness level. The p-value was 0.433.

Still, regarding the participants' employment status, it was noted that no unemployed participant portrayed good knowledge and awareness level, even though 13.33% of the unemployed participants presented moderate knowledge and awareness level, and 86.66% portrayed poor knowledge and awareness level. Consequently, 0.74% of the employed participants showed good knowledge and awareness level, 15.73 presented moderate knowledge and awareness level, and 83.52% portrayed poor knowledge and awareness level. Regarding the students, it was observed that 0.78% showed good knowledge and awareness levels, 14.17% had moderate awareness levels, and 85.03% had poor awareness levels. Lastly, 2.32% of the retired participants presented a good awareness level, 23.25% had a moderate awareness level, and 74.41% portrayed a poor one. The p-value was 0.384 (Table 2).

**Table 2:** Association of demographic data with awareness level

Correlations between the demographic data and the awareness level					
Sex	Good	moderate	poor	Total	P value
Male	4	71	356	431	0.154
Female	0	25	176	201	
Total	4	96	532	632	
Age category	Good	moderate	poor	Total	P value
18-28	2	29	183	214	0.985
29 - 39	1	25	134	160	
40 - 50	1	26	142	169	
51-61	0	14	65	79	
62 and above	0	2	8	10	
Total	4	96	532	632	
Residence	Good	moderate	poor	Total	P value
Mecca	0	13	96	109	0.369
Outside Mecca	4	83	436	523	
Total	4	96	532	632	
Education	Good	moderate	poor	Total	P value
illiterate	0	1	6	7	0.045^*^
Bachelor's degree	1	19	122	142	
General high school / middle school	1	62	362	425	
Master's degree and above	2	14	42	58	
Income	Good	moderate	poor	Total	P value
10000 - 15000 SR	1	11	105	117	0.433
5000 - 10000 SR	1	31	164	196	
Less than 5000 SR	2	28	133	163	
More than 15000 SR	0	26	129	155	
Employment status	Good	moderate	poor	Total	P value
Unemployed	0	26	169	195	0.384
Employed	2	42	223	267	
Retired	1	10	32	43	
Student	1	18	108	127	
Marital status	Good	moderate	poor	Total	P value
Divorced	3	88	447	538	0.237
Married	0	4	39	43	
Widow	1	4	46	51	

Source of information

A larger proportion of the study participants, 45.87%, listed social media as their main source of information on CAE and ADHD. Other sources closely followed this, including relatives and friends at 12.97% and school/university at 7.59%. The other sources of information listed by the participants included awareness campaigns (5.85%), doctors (2.69%), television and newspapers (3.64%), and personal experience (0.63%). Nevertheless, 20.73% of the participants needed information regarding these topics (Table 3).

**Table 3:** Source of information

Source of information		
	Freq	Percent
Social media	290	45.87
School/university	48	7.59
Awareness campaigns	37	5.85
Relative/ friend	82	12.97
Doctors	17	2.69
Television/newspapers	23	3.64
No information about these topic	131	20.73
Personal experience	4	0.63
Total	632	100

## Discussion

ADHD and CAE are different disorders affecting children, and increased awareness and knowledge regarding the conditions can result in better healthcare outcomes and early diagnosis among patients. The present study findings have proven that the awareness level of parents residing in Makkah about ADHD and CAE was significantly poor, corroborating the findings of previous literature [8-10]. For instance, the study conducted by Khalid Al Awad et al. (2022) [10]. "The Extent of Parents' Awareness towards Absence Seizure among Children in Al Baha Region, Saudi Arabia" disclosed that only 30.8% of the study participants had acquired information regarding CAE even though 69.2% did not have any information regarding the condition. Thus, the general evaluation of CAE awareness disclosed that the parents had very poor awareness levels, with a larger proportion of the participants (91.5%) portraying inadequate awareness about CAE and just 1% presenting adequate knowledge regarding the condition. Consequently, Khaled et al. (2017)[9].studied Awareness of the "Saudi Population in Madina Region about Attention Deficit Hyperactive Disorder (ADHD) in Children" and disclosed that the awareness level of ADHD in Madina society was very low, as only 25.1% knew about the condition through experience with ADHD from patients that they know while 14.7% became aware of ADHD by reading on it on medical websites, even as 7.3% became aware of it through social media and print media. A study by Alanazi and Al Turki (2021)[8]. Which focused on "Knowledge and attitude of Attention-Deficit and Hyperactivity Disorder (ADHD) among male primary school teachers in Riyadh City, Saudi Arabia," disclosed that, despite a larger proportion of teachers (76.7%) indicating that they attended ADHD training, only 40.4% indicated that they acquired adequate information regarding the condition. Further, the study findings have disclosed that a larger proportion of the participants comprised females and individuals aged between 18 and 28 years, and this has been attributed to their frequent and increased ease of access to the internet, as well as the observation that internet services are offered to the public at lower rates and the provision and access, is not linked to an individual's income and social status, aspects that corroborate previous study findings conducted in the country [11].

 The female participants were reported to have portrayed good and moderate awareness levels compared to their male counterparts, despite displaying higher rates of poor knowledge levels. Further, participants aged between 18 and 28 presented the highest good and moderate awareness levels compared to participants in other age groups. This was attributable to the observation that, compared to other age groups, individuals aged between 18 and 28 years are highly prone to portray more interest in knowledge acquisition in varied fields, and as a result of increased access to digital information and learning, and also through social media, which has been acknowledged to be the main source of information for the participants. 

The present study has disclosed the existence of a significant correlation (P-value = 0.045) between educational level and the awareness level of ADHD and CAE. For instance, master's degree holders were observed to have the highest rate of good awareness level status (n=2). In contrast, individuals with general high school/middle school education had the highest rate of moderate awareness level (n=62). They were followed immediately by participants with a bachelor's degree education level who portrayed a moderate level of 19 out of 142. In this regard, it can be noted that master's degree holders have solid academic backgrounds and knowledge acquired through their careers, including various scientific research methods and academic appraisal of various research papers and sources. Such academic accomplishments and career experiences have been acknowledged to generate increasingly knowledgeable individuals capable of effectively applying such skills in various situations. This is also a strong indicator of the reason underlying higher ratings regarding good ADHD and CAE awareness levels.

A larger proportion of the younger age participants fell into the general high school/middle school education level category, which clarifies why they were highly rated in the moderate awareness level status category, as previously observed. On the contrary, the bachelor's degree holders were rated second in the moderate awareness level category despite making up the biggest proportion of the study sample, at 67%. The underlying rationale for the rating includes the divergent learning approaches between the two groups, even as the younger generation is increasingly open to accessing and acquiring information outside their fields. Generally, poor awareness level rates were observed in every group, corroborating previous studies' findings [8-10]. For instance, Alanazi and Al Turki (2021) [8].observed that in recent studies conducted in Makkah and Riyadh, the awareness regarding ADHD among elementary school teachers was lower at 58.9% and 17.2%, respectively. Consequently, the study by Khalid Al Awad et al. (2022) [10]. They disclosed that the poor knowledge and awareness among the study participants (parents), particularly about the absence of seizure causes, symptoms/clinical manifestations, and diagnosis, was attributable to the condition's rarity in Saudi Arabia.

Owing to the observation that the target group was mainly individuals/parents residing in Makkah City, for the present study, a four-fold increment in the responses from Makkah was observed compared to responses received from participants living outside Makkah. The researchers discovered no considerable correlations between ADHD and CAE awareness levels and the other demographic data, including age, sex, residence, income, employment status, and marital status. As indicated by the study findings, 84% of parents residing in Makkah City presented poor awareness and knowledge levels, despite the observation that social awareness is a vital aspect impacting early diagnosis of ADHD and CAE in children, leading to improved education performance and general well-being. Such low awareness levels may be improved with future awareness campaigns alongside health awareness programs. Further, the study findings have disclosed that a larger proportion of the participants utilized social media as the main source of information that could be used to spread knowledge regarding ADHD and CAE awareness.

Limitations

The notable limitations of the present study include the observation that it was conducted using an original online questionnaire, which might affect the validity of the responses. Also, there needs to be more previous research and literature regarding these two topics, especially CAE, which carted a need for comparison data. Finally, the study sample size is small compared to the general Saudi population, so the results can only be generalized to some Saudi populations.

## Conclusions

From the outcomes of the present study, one may conclude that the awareness and knowledge about ADHD and CAE among parents residing in Makkah City remain suboptimal. Such poor awareness and knowledge of the two conditions are likely to have a negative effect on children suffering from the condition, their communities and families, and the education and healthcare systems. As such, there is an urgent need to increase and regulate awareness campaigns on ADHD and CAE through mass media and various educational programs. This will enable filling gaps within the community knowledge regarding ADHD and CAE.

The researchers recommend a country-wide study to attain more valid and generalizable results. Further, it is recommended that prospective studies should include larger sample sizes and populations drawn from different parts of Saudi to increase the validity and generalizability of the findings.

## Figures and Tables

**Figure 1:**
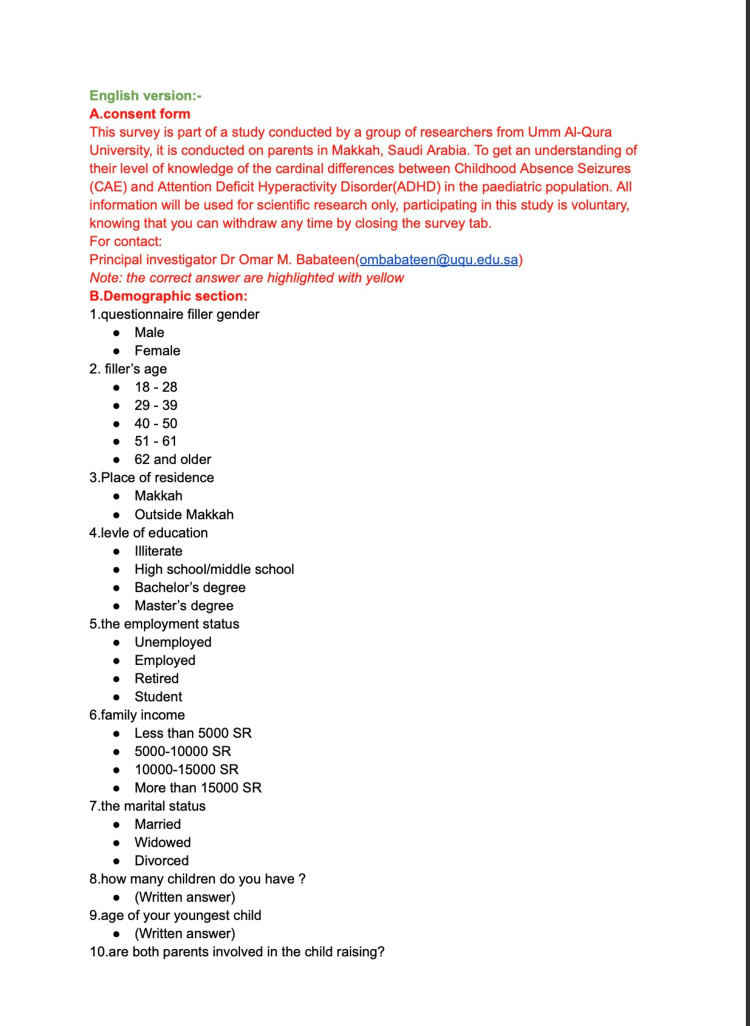
Questionnaire

**Figure 2:**
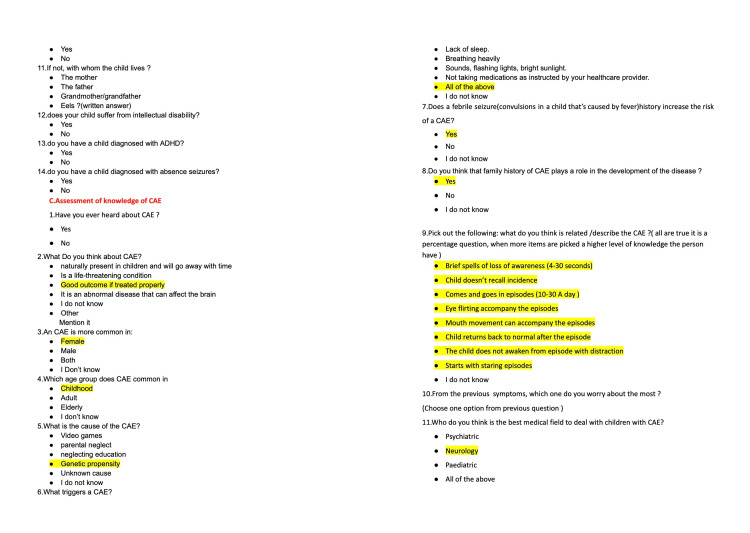
Questionnaire

**Figure 3:**
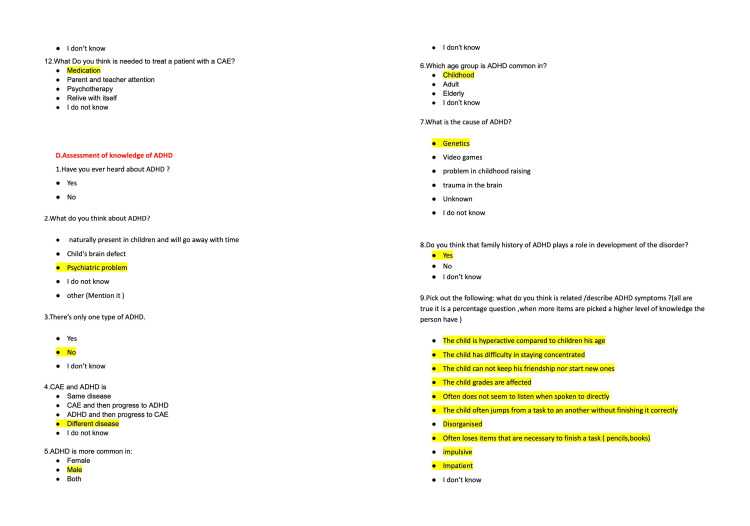
Questionnaire

**Figure 4:**
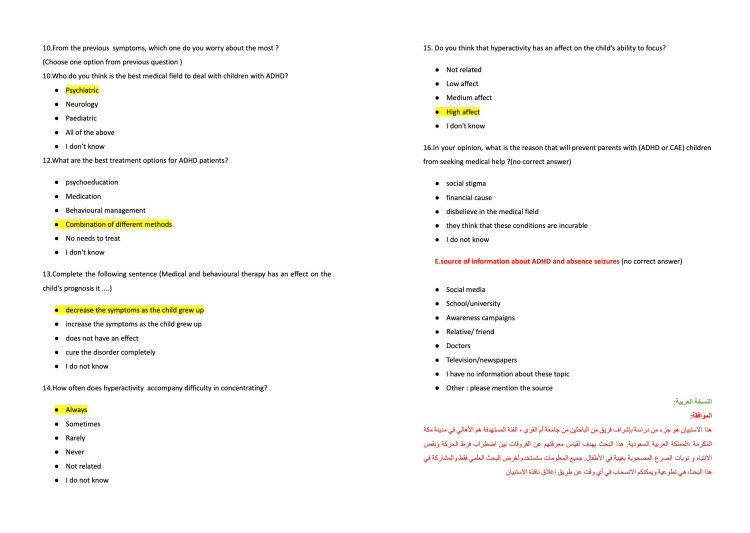
Questionnaire

**Figure 5:**
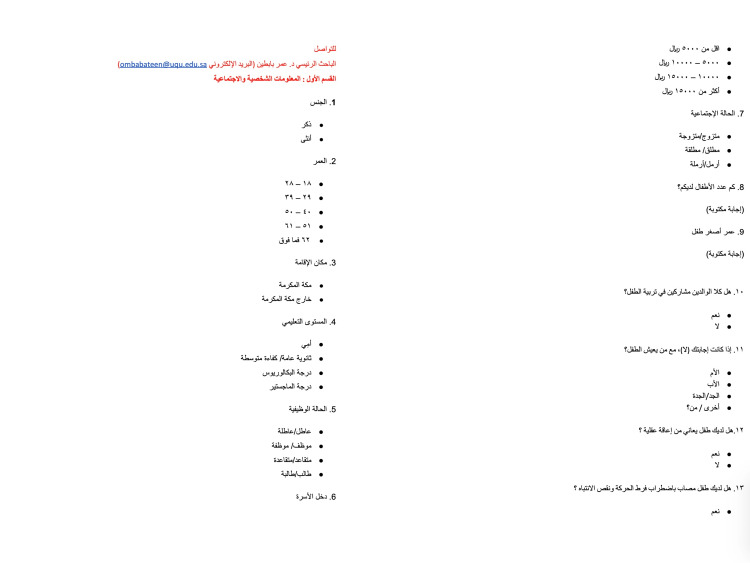
Questionnaire

**Figure 6:**
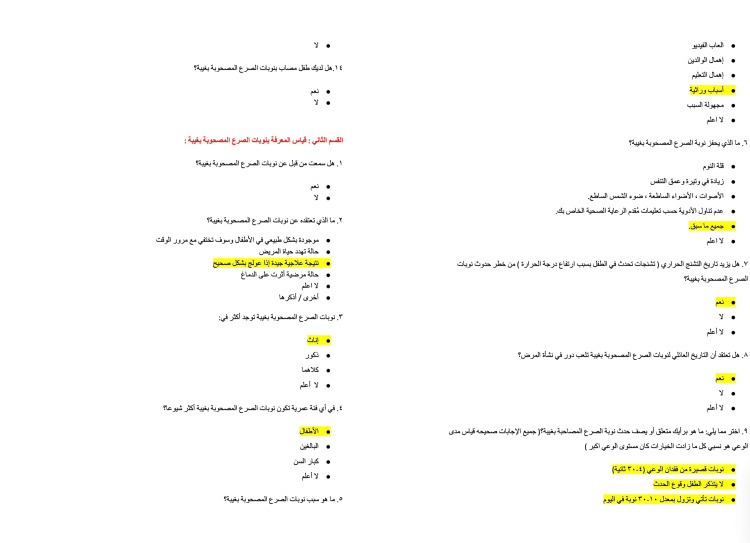
Questionnaire

**Figure 7:**
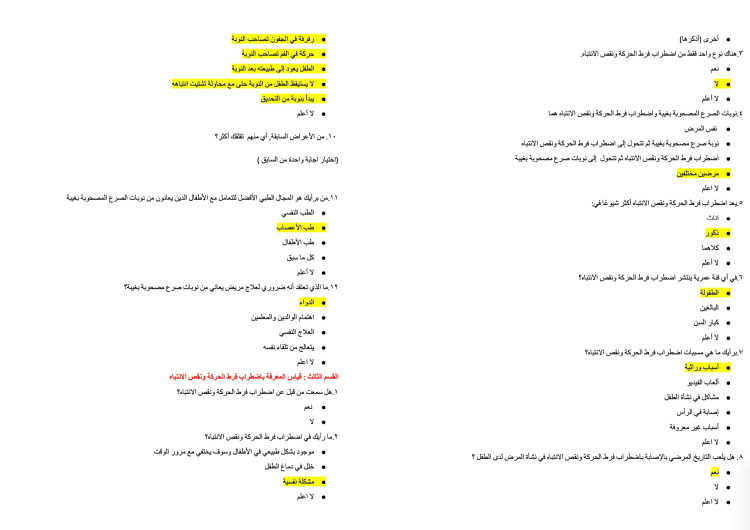
Questionnaire

**Figure 8:**
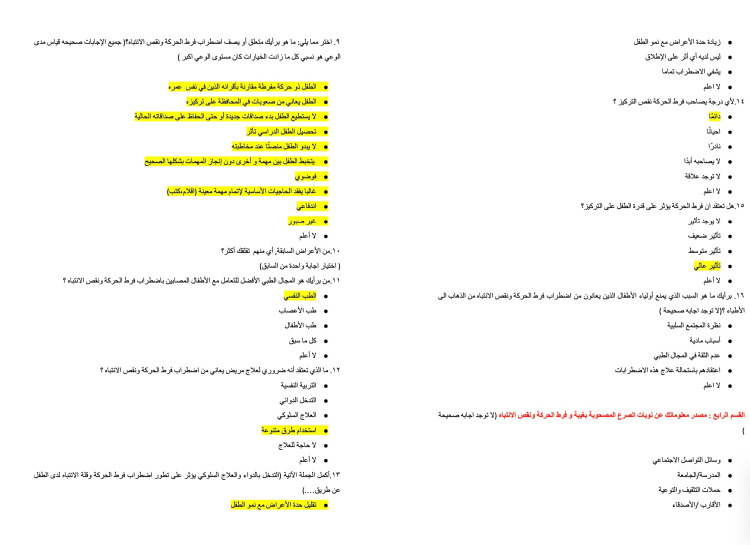
Questionnaire

**Figure 9:**
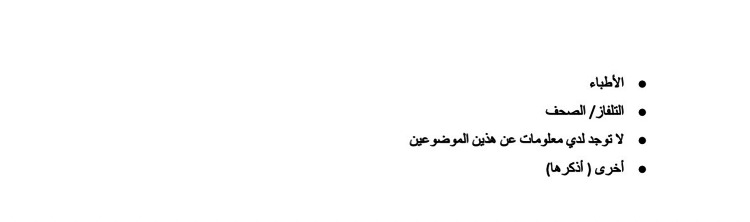
Questionnaire

## Appendices

Questionnaire

QuestionnaireQuestionnaire
